# Diversity of Molecular Mechanisms Conferring Carbapenem Resistance to* Pseudomonas aeruginosa* Isolates from Saudi Arabia

**DOI:** 10.1155/2016/4379686

**Published:** 2016-08-11

**Authors:** Mohamed H. Al-Agamy, Katy Jeannot, Taghrid S. El-Mahdy, Hassan A. Samaha, Atef M. Shibl, Patrick Plésiat, Patrice Courvalin

**Affiliations:** ^1^Pharmaceutics Department, College of Pharmacy, King Saud University, Riyadh, Saudi Arabia; ^2^Microbiology and Immunology Department, Faculty of Pharmacy, Al-Azhar University, Cairo, Egypt; ^3^Laboratoire de Bactériologie, Centre National de Référence de la Résistance aux Antibiotiques, Besançon, France; ^4^Unité des Agents Antimicrobiens, Institut Pasteur, Paris, France; ^5^Microbiology and Immunology Department, Faculty of Pharmacy, Helwan University, Cairo, Egypt; ^6^College of Pharmacy, Al Baha University, Al Baha, Saudi Arabia; ^7^College of Medicine, Alfaisal University, Riyadh, Saudi Arabia

## Abstract

*Background.* This study described various molecular and epidemiological characters determining antibiotic resistance patterns in* Pseudomonas aeruginosa* isolates.* Methods.* A total of 34 carbapenem-resistant* P. aeruginosa* clinical isolates were isolated from samples collected at a tertiary hospital in Riyadh, Saudi Arabia, from January to December 2011. Susceptibility testing, serotyping, molecular characterization of carbapenem resistance, and pulsed-field gel electrophoresis (PFGE) were performed.* Results.* All isolates were resistant to ceftazidime, and more than half were highly resistant (minimum inhibitory concentration (MIC) > 256 mg/L). Fifteen isolates had MIC values ≥64 mg/L for any of the carbapenems examined. Vietnamese extended-spectrum *β*-lactamase (VEB-1) (*n* = 16/34) and oxacillinase (OXA-10) (*n* = 14/34) were the most prevalent extended-spectrum *β*-lactamase and penicillinase, respectively. Verona imipenemase (VIM-1, VIM-2, VIM-4, VIM-11, and VIM-28) and imipenemase (IMP-7) variants were found in metallo-*β*-lactamase producers. A decrease in outer membrane porin gene (*oprD*) expression was seen in nine isolates, and an increase in efflux pump gene (*MexAB*) expression was detected in five isolates. Six serotypes (O:1, O:4, O:7, O:10, O:11, and O:15) were found among the 34 isolates. The predominant serotype was O:11 (16 isolates), followed by O:15 (nine isolates). PFGE analysis of the 34 carbapenem-resistant* P. aeruginosa* isolates revealed 14 different pulsotypes.* Conclusions.* These results revealed diverse mechanisms conferring carbapenem resistance to* P. aeruginosa* isolates from Saudi Arabia.

## 1. Background

*Pseudomonas aeruginosa* is a pathogen emerging as a frequent cause of nosocomial infections, especially pneumonia and sepsis, with mortality rates of 27–48% in critically ill patients [1, 2]. The increasing prevalence of infections caused by multidrug-resistant (MDR)* P. aeruginosa* strains is associated with significant morbidity and mortality [3]. Management of the infections is difficult since strains often display intrinsic and acquired resistance to multiple classes of antibiotics, severely limiting therapeutic options [4]. One feature of* P. aeruginosa* isolates is their high level of intrinsic resistance to a number of antimicrobial agents. The broad-spectrum resistance of these organisms is largely due to low outer membrane permeability [5] and to efflux systems [6]. Moreover, they possess inducible, chromosomally encoded AmpC cephalosporinase belonging to Ambler class C enzymes [7]. Extended-spectrum *β*-lactamases (ESBLs), including TEM, SHV, PER, VEB, GES, OXA-2, and OXA-10 enzymes, are increasingly reported in* P. aeruginosa* [8, 9]. This situation has led to the use of carbapenems as drugs of last resort for treating infections caused by these bacteria. However, the emergence and increasing frequency of isolation of carbapenem-resistant* P. aeruginosa* strains is alarming [10]. Impermeability arising via the loss of outer membrane porin (OprD), upregulation of an active efflux pump (MexAB-OprM), and production of metallo-*β*-lactamases (MBLs) are of great concern [4, 11]. Several types of MBL enzymes, including imipenemase (IMP), Verona imipenemase (VIM), New Delhi MBL (NDM), Seoul imipenemase (SIM), São Paulo MBL (SPM), German imipenemase (GIM), Adelaide imipenemase (AIM), and Dutch imipenemase (DIM), have been identified in* P. aeruginosa* [12]. The genes responsible for the production of MBLs are typically part of class 1 integron structures, which carry other resistance gene cassettes. Hence, isolates producing MBLs are often resistant to different groups of antimicrobial agents, and the resistance can be transferred to various types of bacteria [13, 14]. In this study, we investigated the molecular epidemiology of carbapenem-resistant* P. aeruginosa* isolates obtained from January through December 2011 from patients hospitalized in a tertiary hospital in Riyadh, Saudi Arabia.

## 2. Methods

### 2.1. Bacterial Strains

Thirty-four carbapenem-resistant* P. aeruginosa* isolates were included in this study. The strains were isolated over a one-year period, from January through December 2011, from patients hospitalized in a tertiary hospital in Riyadh, Saudi Arabia. The isolates were identified as* P. aeruginosa* in the clinical laboratory using the VITEK 2 system (bioMérieux, Marcy l'Etoile, France).

### 2.2. Susceptibility Testing

Susceptibility testing to 10 antimicrobial agents, imipenem (IPM), meropenem (MER), doripenem (DOR), ceftazidime (CAZ), amikacin (AN), tobramycin (TM), ciprofloxacin (CIP), colistin (CS), aztreonam (ATM), and ticarcillin (TIC), was performed by an agar dilution method, and the data were interpreted according to the CLSI breakpoints [20].

### 2.3. Serotyping of Isolates

The O-serotypes were determined by a slide agglutination test using four pools (OMA, OMC, OME, and OMF) and 20 monovalent antisera, O1 to O20 (Sanofi Diagnostics Pasteur), according to the manufacturer's recommendations.

### 2.4. MBL Screening

The isolates were screened for MBL production by a double-disk (10 *μ*g of IPM and 2.5 *μ*M ethylenediaminetetraacetic acid) synergy test [20]. A synergistic inhibition zone visible between the two disks indicated a positive result.

### 2.5. Detection of *β*-Lactamase (*bla*) Genes

The primers used in this study are shown in Table 1. The polymerase chain reaction (PCR) amplification of genes for Ambler classes A, B, and D *β*-lactamase enzymes was performed using specific primers for *bla*_GES_, *bla*_VEB_, *bla*_PER_, *bla*_PSE_, *bla*_CTX-M_, *bla*_TEM_, *bla*_SHV_, *bla*_KPC_, *bla*_IMP_, *bla*_VIM_, *bla*_NDM_, *bla*_OXA-I_, and *bla*_OXA-II_ [15–19], followed by sequencing reactions using the Sanger method [21]. Plasmid DNA was extracted according to the Kieser protocol [22].

### 2.6. Pulsed-Field Gel Electrophoresis

Clonal relatedness of the* P. aeruginosa* isolates was evaluated by* Spe*I macrorestriction analysis of genomic DNA, followed by pulsed-field gel electrophoresis (PFGE) [23, 24]. DNA fragments were separated for 20 h at 6 V/cm, 120° included angle, at 14°C using a CHEF-DR II System (Bio-Rad), with the initial and final pulse times of 1 and 35 s, respectively.* P. aeruginosa* strain PAO1 was used as a reference strain. The band patterns that were more than 80% identical were considered related.

### 2.7. RNA Extraction and Real-Time RT-PCR to Measure* oprD* and* mexA* Expression Levels

For RNA isolation, strains were grown in LB broth to the logarithmic phase identified by the optical density at 600 nm, followed by centrifugation. Total RNA was prepared using the TRIzolMax method (Invitrogen, Carlsbad, CA) according to the manufacturer's recommendations. RNase-free DNase (Ambion, Austin, TX) was used to remove DNA. The removal of contaminating DNA was verified by PCR in the absence of reverse transcriptase. Real-time reverse transcription- (RT-) PCR was performed in duplicate using independent RNA extractions and the QuantiTect SYBR Green RT-PCR kit (Qiagen, Inc., Valencia, CA). The primers used for the detection of the* mexA* and* oprD* transcripts are listed in Table 1. Expression levels of the endogenous control gene,* rpsL* (Table 1), were used to normalize the data. A wild-type strain of* P. aeruginosa*, PAO1, was used as a reference [25]. The genes were considered to be up- or downregulated when the amounts of RNA transcripts were at least twofold higher or lower, respectively, than those in PAO1 [26].

## 3. Results

### 3.1. Antibiotic Susceptibility

The 34* P. aeruginosa* strains were highly resistant to TIC, with the minimum inhibitory concentration at which 90% of the isolates were inhibited (MIC_90_) of ≥256 mg/L (Table 2). All isolates were CAZ-resistant (MICs: 32 to >256 mg/L), and 23 of them were highly resistant (MICs > 256 mg/L). All isolates were also resistant to one or more of the carbapenems tested (IPM, MER, and DOR). Thirteen of the 34 isolates (38%) were highly resistant to IPM (MICs ≥ 64 mg/L). ATM resistance was observed in 23 of the 34 isolates (67.65%), and the MIC values ranged from 32 to >512 mg/L. The resistance levels to TM, AN, and CIP were variable. Only one strain was resistant to CS. More than half of the isolates (18 of 34) were resistant to all tested antimicrobials, except CS (Table 2).

### 3.2. Genotyping and Resistance Mechanisms

The genotyping analysis by PFGE was performed for all 34 carbapenem-resistant* P. aeruginosa* isolates, and 14 PFGE profiles (A–N) were identified (Table 2). Five of the 14 clones were represented by 73.5% (*n* = 25) of all isolates. The most common clones were F (*n* = 9), A (*n* = 5), J (*n* = 5), N (*n* = 4), and C (*n* = 2). The other nine clones were represented by one isolate each.

Diverse resistance mechanisms leading to *β*-lactam resistance were identified in the 34 isolates (Table 2). Vietnamese-type ESBLs (VEB-1a and VEB-1b) were found in 16 strains. Oxacillinase- (OXA-10- and OXA-2-) and PSE-type penicillinases were also detected in the present study. Whereas OXA-10 (*n* = 14) was more prevalent, only three strains were found to have OXA-2, and one strain was found to have PSE-1. Two MBL types, VIM and/or IMP, were detected in 12 isolates; each was carried by nine isolates, while seven isolates harbored both VIM and IMP. Five variants of VIM were identified (VIM-1, VIM-2, VIM-4, VIM-11, and VIM-28); however, only one variant of IMP (IMP-7) was detected. All MBL-carrying strains were positive in the MBL phenotypic test, while the test gave negative results for the remaining isolates. Moreover, Guiana ESBL (GES) was detected in three isolates, each producing a different variant of the enzyme (GES-1, GES-4, and GES-6).

Downregulation of OprD porin was detected in nine isolates. In two isolates, this was the only genotypic trait detected, while, in seven other isolates, it was associated with upregulation of the MexAB-OprM efflux pump (two isolates) or with the presence of *β*-lactamase *bla*_VEB-1b_ and *bla*_GES-1_ genes (two isolates), or with both presence of a *β*-lactamase (VEB-1b or PSE-1) and upregulation of MexAB-OprM (three isolates). Nine isolates exhibited only one resistance mechanism. Three of them had an MBL, either VIM or IMP, and were ATM-susceptible but carbapenem-resistant. GES was carried by one isolate, whereas three isolates carried VEB-1b only, and the remaining two isolates only downregulated OprD porin. PCR amplification of *bla*_CTX-M_, *bla*_TEM_, *bla*_SHV_, *bla*_KPC_, and *bla*_NDM_ showed negative results for all isolates.

### 3.3. Serotyping

Six serotypes were identified among the 34 isolates, including O1, O4, O7, O10, O11, and O15 (Table 2). The predominant* P. aeruginosa* serotype was O11 (16/34, 47%), followed by O15 (9/34, 26.5%) and O4 (4/34, 11.7%). Only one strain (number 9) was nontypable by the slide agglutination test. All serotype O15 strains (*n* = 9) were found to have the same pulsotype (F) and the same mechanism of resistance (OXA-10 and VEB-1a). In the present study, 10 isolates were found to harbor a plasmid among the 34 isolates examined for plasmids. The 10 plasmid-carrying isolates produced VIM-28 (clone E, O7, isolate number 8) or OXA-10 and VEB-1a (clone F, O15, isolates numbers 11–19).

## 4. Discussion

The present study clearly demonstrated a high level of resistance to carbapenems in the isolates obtained from patients from a tertiary hospital in Riyadh, Saudi Arabia. Having 16 isolates (47%) with a MIC of ≥64 mg/L for one or more of the carbapenems tested is worrisome. Much lower carbapenem MICs were previously reported in Saudi Arabia [27], with a high prevalence of IPM-resistant (91% of 33 isolates)* P. aeruginosa* isolates, yet none reached the IPM MIC values higher than 32 mg/L. In the above study, two* P. aeruginosa* isolates (6%) were found to be CS-resistant; fortunately, only one (2.9%) CS resistance was observed in our isolates. It should be mentioned that the authors [27] used the CS resistance breakpoint of >2 mg/L in accordance with the CLSI, 2006 guidelines. However, in our study, the resistance breakpoint was >4 mg/L following the CLSI, 2014 recommendations [20]. Consequently, based on this definition, we had one CS-resistant isolate (isolate number 7), which showed a MIC value of 8 mg/L. It is important, when defining resistant strains, to report the reference used for the breakpoint, especially for rare antibiotic resistance phenotypes, such as CS resistance in* P. aeruginosa*.

The diversity of the resistance mechanisms observed in our isolates reveals the different ways by which* P. aeruginosa* can acquire drug resistance. In our study, 12 of the 34 carbapenem-resistant isolates (35.3%) expressed MBLs, either VIM or IMP. This proportion is smaller than that reported in a study from Egypt, in which the carriage rate of MBLs in carbapenem-resistant* P. aeruginosa* isolates was 69%, and most of them were *bla*_VIM-2_ carriers [28]. Furthermore, in two previous studies from Saudi Arabia, MBLs were detected in 41% (16 of 39 isolates) and 60% (15 of 25 isolates) of IPM-resistant isolates, and VIM was found in all MBL-positive isolates [29]. Recently, it was also found in Saudi Arabia that only 11 of 39 carbapenem-resistant* P. aeruginosa* isolates carried carbapenemase genes (*bla*_VIM_ and *bla*_GES-5_), while the rest (28/39, 72%) had no genes to explain the resistance seen [30]. Although carbapenem resistance can also be driven by inactivation of OprD, upregulation of the MexAB-OprM efflux system, or other, unknown, resistance mechanisms, these mechanisms were not investigated in the above study. In our study, 13 of the 34 carbapenem-resistant strains (38%) revealed no known resistance mechanism to explain their resistance since they harbored *bla*_VEB-1_ alone or with *bla*_OXA-10_ or harbored *bla*_GES-6_ alone, and these are not carbapenemase-encoding genes. These strains also produced negative results in the MBL phenotypic test, suggesting other resistance mechanisms responsible for their carbapenem resistance. This finding was also confirmed by the ATM resistance found in all 13 isolates, suggesting that MBLs were not responsible for their carbapenem resistance since MBLs alone do not confer resistance to ATM [12]. It should be noted that transcriptional or posttranscriptional regulation of OprD may also happen leading to loss or reduction of OprD production even if there is no decrease in* oprD* expression (but this is not examined in our study). Additionally, the current study is in accordance with a previous review of Gram-negative bacteria found in Saudi Arabia [31], in which the authors reported that, in* P. aeruginosa*, VEB-like enzymes were most common (in 47% of our isolates), VIM appeared to be the most common MBL (in 29.4% of our isolates), and OXA-10 was frequent (in 44% of our isolates). The present study is the second report of VIM-28, which was first reported in* P. aeruginosa* from Egypt, another Middle Eastern country in the western vicinity of Saudi Arabia [32].

Our findings that all nine serotype O:15 strains (26.5%) belonged to a single pulsotype (F) and all had the same resistance pattern, harboring OXA-10 + VEB-1a, are similar to the data from a previous UK study [33]. The UK study showed that 15 of 32 *bla*_VEB_-positive* P. aeruginosa* isolates (47%) belonged to serotype O:15, had a single PFGE type, carried a VEB-1a variant, but with coexistence of VIM-10, and presented the same resistance pattern. In earlier studies [34, 35], the relationship between* P. aeruginosa* serotypes and antibiotic resistance patterns was investigated, and it was noticed that some serotypes were more associated with resistance to certain antibiotics, although the studies revealed different findings. Additionally, it was also reported that certain serotypes were associated with specific* P. aeruginosa* sequence-type (ST) clones [36, 37]. Guzvinec et al. [37] reported that clones ST235, ST111, and ST132 included serotypes O:11, O:12, and O:6, respectively, and that serotype O:11 was predominant (41% of 103 isolates). In the present study, the O:11 serotype was also the most prevalent serotype (47% of our isolates), although these isolates were found to have diverse PFGE types and different resistance mechanisms.

## 5. Conclusions

The increasing prevalence of infections caused by carbapenem-resistant and MDR* P. aeruginosa* strains is a serious problem, and it is associated with significant morbidity and mortality [3]. The study sets off the alarm of a high level of resistance to carbapenems (47% of isolates with MICs ≥64 mg/L). Further studies of the molecular basis of* P. aeruginosa* resistance in our region are needed to gain a better understanding of the complexity of resistance mechanisms in this organism. The finding that 38% of our isolates revealed no resistance mechanism to explain the carbapenem resistance highlights the urgent demand for more studies to investigate the unknown resistance mechanisms conferring carbapenem resistance to* P. aeruginosa*.

## Figures and Tables

**Table 1:** Primers used in this study.

Gene	Primer	Sequence (5′-3′)	Reference
*bla*_TEM_	MultiTSO-T_for MultiTSO-T_rev	CATTTCCGTGTCGCCCTTATTC CGTTCATCCATAGTTGCCTGAC	[15]

*bla*_SHV_	MultiTSO-S_for MultiTSO-S_rev	AGCCGCTTGAGCAAATTAAAC ATCCCGCAGATAAATCACCAC	[15]

*bla*_CTX-M_	CTX-M-U1 CTX-M-U2	ATGTGCAGYACCAGTAARGTKATGGC TGGGTRAARTARGTSACCAGAAYCAGCGG	[16]

*bla*_VEB_	MultiVEB_for MultiVEB_rev	CATTTCCCGATGCAAAGCGT CGAAGTTTCTTTGGACTCTG	[15]

*bla*_PER_	MultiPER_for MultiPER_rev	GCTCCGATAATGAAAGCGT TTCGGCTTGACTCGGCTGA	[15]

*bla*_GES_	MultiGES_for MultiGES_rev	AGTCGGCTAGACCGGAAAG TTTGTCCGTGCTCAGGAT	[15]

*bla*_VIM_	VIM-F VIM-R	GATGGTGTTTGGTCGCATA CGAATGCGCAGCACCAG	[17]

*bla*_IMP_	IMP-F IMP-R	GGAATAGAGTGGCTTAAYTCTC GGTTTAAYAAAACAACCACC	[17]

*bla*_NDM_	NDM-F NDM-R	GGTTTGGCGATCTGGTTTTC CGGAATGGCTCATCACGATC	[17]

*bla*_KPC_	KPC-Fm KPC-Rm	CGTCTAGTTCTGCTGTCTTG CTTGTCATCCTTGTTAGGCG	[17]

*bla*_OXA-I_ group	OXA-10 F OXA-10 R	TCAACAAAT CGC CAGAGAAG TCC CAC ACC AGA AAA ACC AG	[18]

*bla*_OXA-II_ group	OXA-2 F OXA-2 R	AAGAAACGCTAC TCGCCT GC CCACTCAACCCATCCTACCC	[18]

*bla*_PSE_	PSE-F PSE-R	ACC GTA TTG AGC CTG ATT TA ATT GAA GCC TGT GTT TGA GC	[18]

*mexA*	MexARTF MexARTR2	CAAGCAGAAGGCCATCCTC CGGTAATGATCTTGTCGCCG	[19]

*oprD*	OprDRTF3 OprDRTR3	GAAGCCAAGTACGTGGTCCAG CAGGATCGACAGCGGATAGTC	[19]

*rpsL*	RpsLF1 RpsLR1	GCAACTATCAACCAGCTGGTG GCTGTGCTCTTGCAGGTTGTG	[19]

**Table 2:** Serotypes, resistance patterns, and phenotypic and genotypic characterization of *Pseudomonas aeruginosa* isolates.

Isolate	Serotype	Minimum inhibitory concentration (mg/L)^a^										Genotypic profile	PFGE type	Plasmid
		IPM	MER	DOR	CAZ	CIP	TM	CS	AN	ATM	TIC			
1	O:11	128	64	64	>256	>16	>128	1	128	16	>1,024	IMP-7, OXA-10, VIM-11	A	No
2	O:11	32	64	32	>256	>16	>128	2	128	16	>1,024	IMP-7, OXA-10, VIM-11	A	No
3	O:11	128	128	64	>256	>16	128	1	32	16	>1,024	IMP-7, OXA-10, VIM-11	A	No
4	O:11	>256	>128	>128	>256	>16	64	1	16	16	>1,024	IMP-7, OXA-10, VIM-2	B	No
5	O:11	>256	>128	>128	128	0.5	>128	2	32	8	>1,024	VIM-4, IMP-7, OXA-2	C	No
6	O:11	>256	>128	>128	256	0.5	1	1	64	8	>1,024	VIM-4, IMP-7, OXA-2	C	No
7	O:11	>256	>128	128	128	>16	>128	8	256	32	>1,024	VIM-4, IMP-7, OXA-2	D	No
8	O:7	64	64	32	256	>16	>128	2	128	8	>1,024	VIM-28	E	>50 kb
9	NT	128	128	64	>256	>16	64	2	>256	>512	>1,024	OXA-10, VEB-1a, GES-4, VIM-1	A	No
10	O:4	16	16	4	>256	>16	64	2	>256	128	>1,024	IMP-7, OXA-10, VIM-11	A	No
11	O:15	32	32	32	>256	>16	128	2	64	>512	>1,024	OXA-10, VEB-1a	F	50 kb
12	O:15	32	32	>128	>256	>16	128	2	32	512	1,024	OXA-10, VEB-1a	F	50 kb
13	O:15	32	32	16	>256	>16	>128	2	128	>512	>1,024	OXA-10, VEB-1a	F	50 kb
14	O:15	32	32	16	>256	>16	64	1	32	>512	>1,024	OXA-10, VEB-1a	F	50 kb
15	O:15	32	32	8	>256	>16	64	1	32	>512	1,024	OXA-10, VEB-1a	F	50 kb
16	O:15	32	32	16	>256	>16	128	2	64	>512	>1,024	OXA-10, VEB-1a	F	50 kb
17	O:15	32	128	64	>256	>16	64	1	32	>512	>1,024	OXA-10, VEB-1a	F	50 kb
18	O:15	>256	128	64	>256	>16	64	2	32	>512	>1,024	OXA-10, VEB-1a	F	50 kb
19	O:15	32	32	16	>256	>16	128	2	32	>512	>1,024	OXA-10, VEB-1a	F	50 kb
20	O:11	128	128	128	>256	>16	64	1	32	16	>1,024	IMP-7	G	No
21	O:11	16	32	32	>256	>16	64	1	16	8	512	IMP-7	H	No
22	O:11	16	32	16	>256	>16	32	2	16	>512	>1,024	VEB-1b, OprD−, MexAB+	I	No
23	O:11	16	16	4	>256	>16	64	2	64	>512	1,024	VEB-1b, OprD−, MexAB+	J	No
24	O:11	16	16	8	>256	>16	64	2	32	512	1,024	VEB-1b	J	No
25	O:11	>256	32	16	>256	>256	64	2	64	256	>1,024	VEB-1b, OprD−	J	No
26	O:11	16	16	4	>256	>16	32	2	32	>512	>1,024	VEB-1b	J	No
27	O:11	13	8	4	>256	>16	32	2	32	512	1,024	VEB-1b	J	No
28	O:4	32	32	2	128	4	128	1	16	16	>1,024	GES-1, OprD−	K	No
29	O:4	128	128	64	256	>16	64	2	>256	32	1,024	GES-6	L	No
30	O:10	16	16	8	32	0.3	1	2	4	512	256	OprD−	M	No
31	O:4	32	16	8	64	0.5	1	1	4	64	256	OprD−, MexAB+	N	No
32	O:10	8	8	8	64	0.3	1	2	2	64	64	OprD−	N	No
33	O:11	64	32	32	64	1	1	1	8	64	128	OprD−, MexAB+	N	No
34	O:1	32	16	4	8	1	64	2	8	16	>1,024	OprD−, PSE-1, MexAB+	N	No

^a^Minimum inhibitory concentration interpretation according to CLSI (2014) recommendations [20].

IPM: imipenem; MER: meropenem; DOR: doripenem; CAZ: ceftazidime; AN: amikacin; TM: tobramycin; CIP: ciprofloxacin; CS: colistin; ATM: aztreonam; TIC: ticarcillin.

NT: nontypable.

OprD−: downregulation of OprD porin.

MexAB+: upregulation of MexAB.

## Abbreviations

AIM: Adelaide imipenemase
AN: Amikacin
ATM: Aztreonam
*bla*: Beta-lactamase gene
CAZ: Ceftazidime
CIP: Ciprofloxacin
CS: Colistin
DIM: Dutch imipenemase
DOR: Doripenem
ESBL: Extended-spectrum *β*-lactamase
GES: Guiana extended-spectrum *β*-lactamase
GIM: German imipenemase
IMP: Imipenemase
IPM: Imipenem
MBL: Metallo-*β*-lactamase
MDR: Multidrug-resistant
MER: Meropenem
MIC: Minimum inhibitory concentration
NDM: New Delhi metallo-*β*-lactamase
Opr: Outer membrane porin
OXA: Oxacillinase
PCR: Polymerase chain reaction
PFGE: Pulsed-field gel electrophoresis
RT: Reverse transcription
SIM: Seoul imipenemase
SPM: São Paulo metallo-*β*-lactamase
ST: Sequence-type
TIC: Ticarcillin
TM: Tobramycin
VEB: Vietnamese extended-spectrum *β*-lactamase
VIM: Verona imipenemase.
